# The Impact Assessment of CuO Nanoparticles on the Composition and Ultrastructure of *Triticum aestivum* L.

**DOI:** 10.3390/ijerph18136739

**Published:** 2021-06-23

**Authors:** Ildiko Lung, Ocsana Opriş, Maria-Loredana Soran, Otilia Culicov, Alexandra Ciorîță, Adina Stegarescu, Inga Zinicovscaia, Nikita Yushin, Konstantin Vergel, Irina Kacso, Gheorghe Borodi, Marcel Pârvu

**Affiliations:** 1 National Institute for Research and Development of Isotopic and Molecular Technologies, 67-103 Donat, 400293 Cluj-Napoca, Romania; ildiko.lung@itim-cj.ro (I.L.); ocsana.opris@itim-cj.ro (O.O.); loredana.soran@itim-cj.ro (M.-L.S.); alexandra.ciorita@itim-cj.ro (A.C.); adina.stegarescu@itim-cj.ro (A.S.); irina.kacso@itim-cj.ro (I.K.); gheorghe.borodi@itim-cj.ro (G.B.); 2 Frank Laboratory of Neutron Physics, Joint Institute for Nuclear Research, 6 Joliot-Curie, 141980 Dubna, Russia; inga@jinr.ru (I.Z.); ynik_62@mail.ru (N.Y.); verkn@mail.ru (K.V.); 3 National Institute for Research and Development in Electrical Engineering ICPE-CA, 313 Splaiul Unirii, 030138 Bucharest, Romania; 4 Faculty of Biology and Geology, Babeș-Bolyai University, 5-7 Clinicilor, 400006 Cluj-Napoca, Romania; marcel.parvu@ubbcluj.ro; 5 Horia Hulubei National Institute for Physics and Nuclear Engineering, 407 Atomistilor, 077125 Magurele, Romania

**Keywords:** wheat, nanoparticles, polyphenols, assimilating pigments, antioxidant capacity, elemental content

## Abstract

In the present study, the effects of copper oxide nanoparticles (CuO NPs) on bioactive compounds, the ultrastructural modifications which can occur, and elemental content of wheat were investigated. Changes in the wheat plants grown in presence or absence of CuO NPs were estimated. The application of CuO NPs decreased the amounts of chlorophylls and carotenoids and increased the amounts of polyphenols and antioxidant capacity. Ultrastructural analysis showed that the plants treated with CuO NPs were negatively affected. Soil amending completely inhibited the accumulation of seventeen elements, while K, Br, Al, and Zn were accumulated and Cl, Na, Ba, and Sr content decreased in wheat samples, regardless of the type of NPs applied. The application of chemically obtained NPs induced the most significant changes, completely blocking the assimilation of Fe, Mo, As, Sb, and Sm, and favoring much higher accumulation of Br than biogenic NPs. The decrease in chlorophylls and carotenoids is correlated with increase in antioxidant capacity, and occurs with increase of Mo, Al, Mg, K, Zn, and Ca content. The behavior of total polyphenols is correlated with Br content, and antagonist to Al behavior. From the point of view of bioactive compounds, the most affected plants were those that grew in the presence of CuO-NP-cel, while from the point of view of elementary analysis, the most affected plants were those grown in the presence of CuO-NP. By corroborating the obtained results, it was found that the CuO NPs have a negative effect on wheat plants.

## 1. Introduction

Multiple applications of nanoparticles (NPs) in various areas of industry and science, such as medicine, pharmacology, electronics, biology, or agriculture, are directly related to their release into environmental multimedia [1,2,3]. Products contain metal NPs (e.g., sunscreens, lotions, or cosmetics), which increases the risk of environmental exposure after manufacture processes or after human use. Because the wastewater can easily end up in soil, this could be an important cause of NP accumulation in the environment [4]. Due to this increased load of NPs, there is a concern about their interaction with flora and fauna, due to the significant toxic effects that may occur. In particular, metal oxide NPs, such as CeO_2_, CuO, TiO_2_, and ZnO, are progressively incorporated into agricultural products, including fertilizers, additives for soil remediation, growth regulators, pesticides, herbicides, or disposal of waste and polluted water [5], while the metal NPs, such as Ag and Au, are used in many commercial products [6].

Copper NPs have been used as disinfectant for wastewater and are more active than copper oxide NPs [7]. These NPs are used in agriculture as pesticide and fungicide components for plants’ protection against bacterial and fungal infections. In addition, Cu is a microelement present in plants in different quantities. Various studies indicate negative, positive, or neutral effects of metal NPs on growth and seed germination of higher plants [8,9,10,11,12]. Due to the physiological and morphological diversity of plants, the ability to uptake NPs varies from one plant to another [13,14,15]. Thus, there may be different mechanisms by which NPs accumulate inside plant tissues [16]. For Cu NPs, several mechanisms have been described, but none have been confirmed by now [6].

The impact of long-term administration of CuO NPs (50 and 500 mg/kg) on wheat growth was studied by Wanga et al. [17]. The addition of CuO NPs inhibited the growth and development of wheat grains and reduced the levels of some essential amino acids in wheat grains. In addition, Adams et al. [18] studied the effect of CuO NPs (>10 mg Cu/kg) on wheat grown in sand. They found that inhibition of root elongation and proliferation of root hair caused by CuO NPs was due to the release of Cu from dissolution at the root surface.

In order to distinguish the adsorption and uptake of CuO NPs on plant root surface, a study was conducted in which different cations, surfactant, and metal complexing agents were used for desorbing CuO NPs. It was found that most of the CuO NPs that adsorbed on the wheat root surface strongly interacted with the root surface, some of them being mechanically adhered [19].

Other authors evaluated the influence of the solubility of Cu-based NPs on Cu uptake and NP association with plant roots in wheat plants. The results obtained from their study showed that NPs, by association with the roots, could play a potential role in slow administration of micronutrients in the plant [20]. Analysis of the CuO NPs effects on rice showed that seed germination was significantly reduced and that the root cells were damaged along with an increase in H_2_O_2_, an accumulation of proline, and a decrease in carotenoid levels [21].

Recently, the interest in NPs obtained by green synthesis, including those that use plant extracts, has increased [22,23,24]. Even if the synthesis of these NPs reduces the amount of synthetic reagents, the toxicity of the obtained NPs must be low. Recent studies have shown that the application of inorganic NPs may ameliorate the undesirable effects of the accumulation of potentially toxic elements (PTEs) [25,26]. The use of NPs may also reduce the content of some essential elements for plant nutrition such as Ca, N, P, S, Fe, Na, and K, or Zn and Mn, which may be both essential and toxic [27,28]. Different nanomaterials prepared by green synthesis have different applications in agriculture, and the study of their toxicity is very important. Therefore, from an ecological perspective, the understanding of the toxicity of CuO NPs and their effects toward environmentally relevant plant species is of great importance.

Thus, in the present study, wheat was selected due to its extensive use as a crop and animal food, with important nutritional values. The importance of wheat in food production is undeniable. Wheat is grown on more land area than any other food crop, and the world trade in wheat is greater than for all other crops combined. Although the worldwide annually harvested area has slightly varied around 215 Mha since 1994, the world production of wheat has increased about 1.5 times in the same period [29].

The main objective of this study is to evaluate the effects of chemically- and green-synthesized CuO NPs on *Triticum aestivum* L. plants. The modification of plant ultrastructure, bioactive compounds concentration, and their elemental content were taken into account to assess the NPs impact on the plant growth. The green-synthesized NPs were obtained using celandine (*Chelidonium majus* L.) extract, respectively, blackthorn (*Prunus spinose* L.) extract. The plant materials extracts, rich in antioxidant phytochemicals, act as reducing and capping agents, leading to the formation of metallic NPs [30,31].

## 2. Materials and Methods

### 2.1. Chemicals and Materials

For NP synthesis, copper sulphate (CuSO_4_·5H_2_O) and ascorbic acid (AA) were purchased from Alfa Aesar, Kandel, Germany, while cetyltrimethylammonium bromide (CTAB) was procured from Reactivul Bucuresti, Romania. For pH adjustment, sodium hydroxide (NaOH) was acquired from Sigma Aldrich, Taufkirchen, Germany. *Chelidonium majus* L. (celandine) was collected from “Alexandru Borza” Botanical Garden of Cluj-Napoca (46°45′36″ N and 23°35′13″ E), Romania, and was identified by Dr. Marcel Pârvu, Babes-Bolyai University of Cluj-Napoca. A voucher specimen (CL 663 692) was deposited at the Herbarium of Babes-Bolyai University of Cluj-Napoca [32]. Blackthorn (*Prunus spinosa* L.), an organic product from organic farms containing 100% fruit, was procured from the local naturist shop (producer: Dary Natury, *Grodzisk* Wielkopolski, Poland; importer: SC Bio Boom Company SRL, Alexandria, Romania).

For total polyphenols quantification and antioxidant capacity determination, Folin–Ciocalteu reagent, gallic acid, anhydrous carbonate 2,2′-diphenyl–picrylhydrazyl (DPPH), and 6–hydroxy-2,5,7,8-tetramethylchroman–2 carboxylic acid (Trolox) were employed from Sigma-Aldrich, Taufkirchen, Germany. The ethanol (96%) used for extractions was purchased from Chimreactiv SRL, Bucuresti, Romania.

All chemicals used in the experiments were of analytical grade, and the ultrapure water was produced with a Milli-Q purification system (Millipore, Bedford, MA, USA).

### 2.2. Plant Extract and Nanoparticle Preparation

#### 2.2.1. Extract Preparation

Celandine extract: Plant extract preparation and chemical composition are previously described [32]. Briefly, fresh herb was extracted with 60% ethanol in the Mycology Laboratory of Babes-Bolyai University by cold repercolation method at room temperature for three days. The celandine extract contained 1 g plant material in 1 mL 30% ethanol (*w*/*v*) and was stored at 4 °C for further use.

Blackthorn extract: Over 1 g of the dried and finely ground blackthorn fruits was added to 10 mL of ethanol:water (40:60, *v*/*v*). The mixture thus obtained was sonicated (Transsonic T 310 at 35 kHz and installed power of 95 W) for 10 min at 60 °C. The next stage of ultrasound was centrifugation (12,052× *g*, for 10 min) and decanting of the obtained extract. Until the time of analysis, the extract was stored at 4 °C.

#### 2.2.2. Nanoparticle Synthesis

Three types of CuO NPs were obtained in this study. The first type of CuO NP was obtained according to the modified method used by Kathad and Gajera [33]. A total of 0.04 M of CuSO_4_·5H_2_O and 0.055 M of AA were dissolved in 100 mL of ultrapure water in a 250 mL flat-bottom flask. The mixture was stirred on a magnetic plate, then 0.015 M CTAB was added to solution at room temperature, and then another 50 mL of water. The pH of the mixture was brought to 6.5 with the help of NaOH, after which it was heated to 85 °C and stirred at 530 rpm until the red color of the brick was obtained. After obtaining this color, the mixture was stirred for another 15 min and then was cooled with water. NPs were separated by centrifugation, washed with ultrapure water and ethanol, and dried in the oven at 65 °C.

The second type of CuO NP was obtained by mixing 0.08 M CuSO_4_·5H_2_O dissolved in 16 mL of ultrapure water with 8 mL of celandine extract, and the same steps were followed as above.

The third type of NP was obtained identical to the second type, except that in this case, blackthorn extract was used.

The CuO NPs considered for the present study were abbreviated as follows: NPs synthesized by chemical method (CuO-NP), NPs biologically synthesized using celandine extract (CuO-NP-cel) or blackthorn extract (CuO-NP-bth).

#### 2.2.3. Characterization of NPs

The NPs synthesized were characterized by different techniques. The structural characterization of the NPs was performed by X-ray powder diffraction analysis using a D8 Advance diffractometer (Rigaku, Tokyo, Japan) equipped with Ge (111) monochromator, CuKα1 radiation, and LynxEye superspeed position detector. The aspects of the NPs obtained were analyzed using scanning/transmission electron microscope, STEM HITACHI HD2700 (HITACHI, Tokyo, Japan), from LIME-INCDTIM, Cluj-Napoca, Romania, cold field emission, operated at 200 kV and coupled with double cut EDX (energy-dispersive X-ray spectroscopy) system, operated at 10^−7^ pressure. It was used to confirm the elemental distribution in the NPs, and the size distribution was analyzed using Image J (version Java 8). FTIR measurements were performed with a JASCO 6100 FTIR spectrometer (JASCO Deutschland GmbH, Pfungstadt, Germany) in the 4000–400 cm^−1^ spectral domain with a resolution of 4 cm^−1^ by using the KBr pellet technique [34].

### 2.3. Plant Growth Conditions

ANDRADA autumn wheat (*Triticum aestivum* L.) was used to study the adsorption and uptake of synthesized NPs. Wheat seeds (50 grains at developmental stage 00 according to the BBCH-scale) were sown at a depth of 1 cm in plastic pots (0.81 L, 13.5 cm in diameter) containing 400 g of garden substrate with active humus and fertilizer for 6 weeks (Agro CS, Veľké Dravce, Slovakia, 50 L.). The physicochemical characteristics of the soil used were pH = 5.5 ± 0.5, N—at least 0.1 m/m%, P_2_O_5_—at least 0.01 m/m%, and K_2_O—at least 0.03 m/m%. The plants were kept in a growth chamber (capacity 256 L, Memmert GmbH, Büchenbach, Germany) under controlled light conditions (for 12 h from 24 h), 60% humidity, and a day/night temperature cycle of 20/10 °C. The experiment took place during one season. For each experiment, including the control group, three independent pots were prepared.

Control wheat was sown only in garden substrate. The plants on which the influence of CuO NPs was studied were sown in garden substrate mixed with 120 mg chemically- and green-synthesized NPs (NP/soil ratio 3:10,000). Watering was performed every two days with 80 mL of water. At stage 19, the plants were harvested and prepared for further analyses.

### 2.4. Analysis of Plant Tissues and Soil after Harvesting

#### 2.4.1. Extraction and Characterization of Assimilating Pigments

In order to obtain the extract, using the method of Opris et al. (2020) modified [35], 20 mL of acetone were added to the minced wheat and stirred on a shaker for 30 min at 300 rotations per minute, at room temperature, then centrifuged for 10 min, and the supernatant was decanted. The operation was repeated with another 20 mL, respectively, 10 mL acetone, the final mixture of wheat and acetone being in a ratio of 1:100. The solutions came together in the same bottle. Extraction of pigments was performed in triplicate.

Qualitative but also quantitative analysis of chlorophyll a, chlorophyll b, and carotenoids in a plant extract can be performed using UV–VIS spectroscopy. In this respect, the absorption spectra of the extracts in the wavelength range between 400 and 750 nm were recorded. The concentrations for chlorophyll a (C_a_), chlorophyll b (C_b_), and total carotenoids (C_(x+c)_) were calculated from the following formulas [36]:

(1) $$C_{a}\left(\mathrm{mg}/\mathrm{mL}\right)=11.24*A_{661.6}-2.04*A_{644.8}$$

(2) $$C_{b}\left(\mathrm{mg}/\mathrm{mL}\right)=20.13*A_{644.8}-4.19*A_{661.6}$$

(3) $$C_{\left(x+c\right)}\left(\mathrm{mg}/\mathrm{mL}\right)=\left(1000*A_{470}-1.90*C_{a}-63.14*A_{b}\right)/214$$

#### 2.4.2. Extraction and Spectrophotometric Analysis of Total Polyphenols

The polyphenolic extracts were obtained from the fresh wheat plant. In order to obtain the alcohol-based extract, 60% ethanol was added over the milled wheat, the final mixture of wheat and ethanol being 1:15. The mixture was sonicated for 30 min at room temperature and then centrifuged for 10 min at 7000 rpm, and the supernatant was decanted and stored at 4 °C until analysis [35].

The total polyphenol content was determined by the Folin–Ciocalteu method [37], which is based on the chemical reduction of the Folin–Ciocalteu reagent. The products resulting from the reduction of the metal oxides give a blue colored compound having a wide absorption band with a maximum at 765 nm. For this purpose, 1 mL of the extract was added to a 10 mL graduated flask containing 5 mL of ultrapure water. Over 0.5 mL of Folin–Ciocalteu reagent was added, and the contents were mixed. After 3 min, 1.5 mL of Na_2_CO_3_ concentration 5 g/L was added, and it was brought to 10 mL with ultrapure water. After keeping the samples at 50 °C (in a water bath) for 16 min in closed flasks, followed by cooling to room temperature, the absorbances were read relative to the blank sample (ultrapure water) at the wavelength of 765 nm. All measurements were taken in triplicates, and mean values were calculated.

The total polyphenol concentration of the samples to be analyzed was calculated using the standard curve, performed under the same conditions as the sample solutions. In order to draw the calibration curve, a standard solution of gallic acid 1 mg/mL was employed, from which solutions for the range of 0.002–0.8 mg/mL were prepared by successive dilutions [38]. The linear equation of the standard calibration curve was y = 0.5865x + 0.0059 (R^2^ = 0.9991).

#### 2.4.3. Determination of Antioxidant Capacity

Antioxidant capacity was investigated by 2,2-diphenylpicrylhydrazyl radical scavenging activity (DPPH) method, reported by Brand-Williams et al. [39] and slightly modified. Thus, 0.01 mL of the alcoholic extract was added to 3.9 mL of DPPH radical solution (0.0025 g/100 mL methanol) and after 10 min remaining in the dark, the absorbance of the mixture was measured at 515 nm and compared to the control sample (0.01 mL extract added to 3.9 mL methanol). The results were calculated from the calibration curve drawn for different solutions of Trolox (0–400 µM) and expressed in mM Trolox/100 g plant. The linear equation of the standard calibration curve was y = 0.2003x + 0.0119 (R^2^ = 0.9993). All determinations were made in triplicate.

#### 2.4.4. TEM Analysis of Wheat Tissue

The leaves and roots of wheat were collected at the same time, and immediately after harvest, they were prepared for transmission electron microscopy (TEM) analysis as previously indicated [40]. Briefly, the samples were placed in 2.7% glutaraldehyde for 1 h, thoroughly washed with phosphate-buffered saline (PBS), fixed with OsO_4_, and washed again in PBS. After the fixation step, the samples were dehydrated in acetone, infiltrated and embedded in epoxy resin Epon 812, and polymerized at 60 °C for three days.

Ultrathin sections were obtained using a Diatome diamond knife (DiATOME, Hatfield, PA, USA) and Leica UC7 ultramicrotome (Leica Microsystems, Wetzlar, Germany; from the Integrated Electron Microscopy Laboratory—LIME, INCDTIM, Cluj-Napoca, Romania). The sections were double-stained with uranyl acetate and lead citrate and further analyzed at TEM Jeol JEM 1010 (JEOL, Tokyo, Japan, from “Constantin Craciun” Electron Microscopy Laboratory, “Babes-Bolyai” University, Cluj-Napoca, Romania).

An elemental analysis was also conducted on the samples using scanning electron microscope (SEM) HITACHI SU8230 (HITACHI, Tokyo, Japan) cold field emission, operating at 30 kV and coupled with a double EDX detector (LIME-INCDTIM, Cluj-Napoca, Romania).

#### 2.4.5. Elemental Content of the Wheat Biomass and Soil Substrate

To determine the elemental content of the wheat biomass and soil substrate, neutron activation analysis (NAA) at the pulsed fast reactor IBR-2 (FLNP JINR, Dubna, Russia) was used. The description of the irradiation channels and the pneumatic transport system REGATA of the IBR-2 can be found elsewhere [41]. To determine short-lived isotopes (Al, Ca, Cl, Cu, Mg, Mn, Ti, V), biological (about 0.3 g) and substrate samples (about 0.1 g) were irradiated for 3 min and 1 min, respectively, under a thermal neutron fluency rate of approximately 1.6 × 10^13^ n cm^−2^ s^−1^. Both types of samples were measured for 15 min. In the case of long-lived isotopes (As, Au, Ba, Br, Ce, Co, Cr, Cs, Eu, Fe, Hf, K, La, Mo, Na, Ni, Rb, Sb, Sc, Sm, Sn, Sr, Ta, Tb, Tm, U, W, Yb, Zn, Zr), the biological (about 0.3 g) and substrate (about 0.1 g) samples were irradiated for 4 days under a resonance neutron fluency rate of approximately 3.31 × 10^12^ n cm^−2^ s^−1^, repacked, and measured using high-purity germanium detectors twice (after 4–5 days and 20–23 days of decay). More information about irradiation of biological and substrate samples can be found in Culicov et al. [42]. The NAA data processing and determination of element content was performed using the software developed at FLNP JINR [43].

High quality of the analysis was ensured through the use of certified standards: Trace Elements in Soil (2709), PINE NEEDLES (1575a), CALCAREOUS SOIL (690CC), MARINE SEDIMENT (433) and Trace Elements in Coal (1632c). The reference materials were irradiated in the same conditions with samples. The measured concentrations and certificates values are in good agreement. NAA results presented in the paper are expressed on a dry weight basis.

### 2.5. Data Analysis

Data from the study are presented as the mean of three replicates ±SD. The differences between groups were tested for significance using one-way ANOVA followed by Tukey’s test using ORIGIN 9 (Origin Lab Corporation, Northampton, MA, USA). Statistical significance was considered at 95% confidence intervals. Moreover, principal component analysis (PCA) was performed using Minitab 17 (Minitab Ltd., Coventry, UK).

Exploratory analysis methods were used to study and present the data regarding the elemental content of the samples. Three parameters were calculated in order to quantitatively characterize the origin and transfer of the elements [44,45]:

1. Mobility ratio or transfer factor that expresses the ratio of element concentration in wheat to its concentration in soil; MR = Cwheat/Csoil.
2. Enrichment factor (EFsoil), relative abundance of a chemical element in a soil compared to the relative abundance with respect to control sample; EFsoil = (C/Al)soil/(C/Al)soil control.
3. Enrichment factor of plant (EFwheat), calculated as EFwheat = (C/Al)wheat/(C/Al)control wheat.

## 3. Results and Discussion

### 3.1. NP Characterization

#### 3.1.1. XRD Analysis

**Figure 1:**
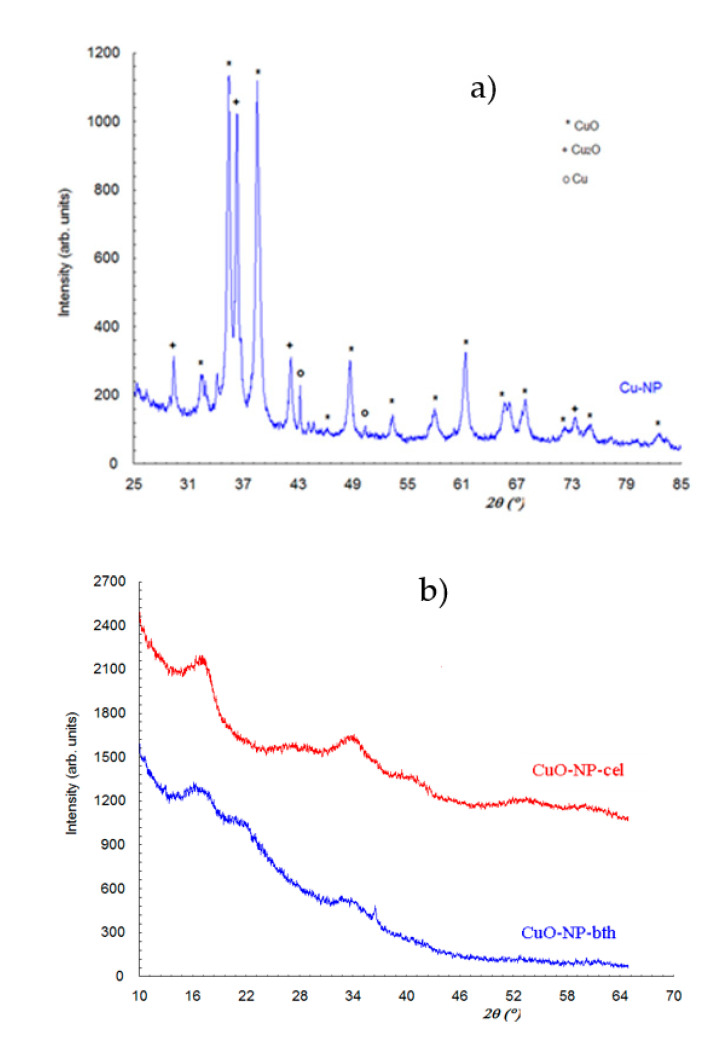
X-ray powder diffraction pattern for (**a**) chemically- and (**b**) green-synthesized NPs.

To determine the main Cu phases that occurred during the formation of NPs, X-ray diffraction (XRD) analyses were performed. Thus, the X-ray powder diffraction pattern for CuO-NP (Figure 1) contains three phases, namely monoclinic CuO phase (PDF:65-2309), cubic Cu_2_O phase (PDF:65-3288), and cubic Cu phase. Crystallite size evaluated by Scherrer relationship was about 230 Å for CuO, 280 Å for Cu_2_O, and 500 Å for Cu. From the width of the diffraction peaks, it is seen that all the phases present in the sample are of the order of tens of nanometers, so they are in the nano domain.

**Figure 2:**
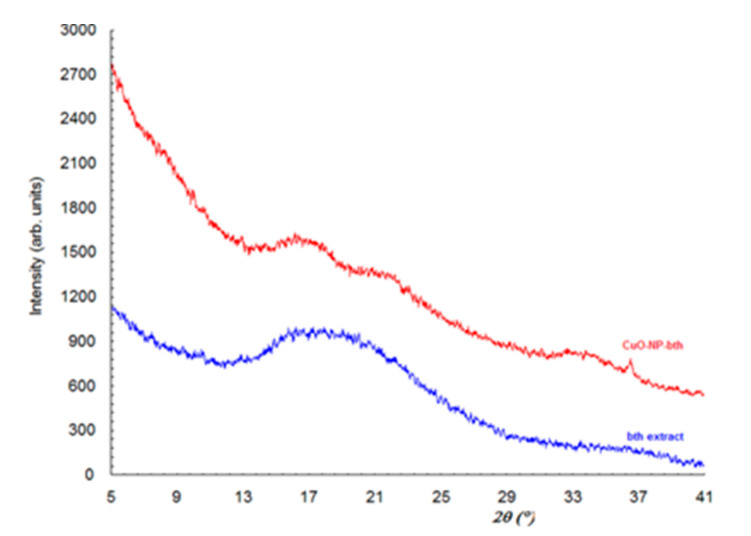
X-ray powder diffraction patterns of CuO-NP-bth and blackthorn extract.

From X-ray powder diffraction for Cu-Np (Figure 1a), the result is that the sample contains CuO and Cu_2_O as the majority phase and Cu as the minority phase. The X-ray powder diffraction for CuO-NP-bth and CuO-Np-cel (Figure 1b) highlights the amorphous character of these samples. It is observed that in both samples there are two very broad diffraction maxima (halos characteristic of amorphous phases) of higher intensity at angles 16.600 and 33.700, respectively. In the Cu-Np-bth sample there is also a halo of lower intensity at the angle 21.40, and in the Cu-O-Np sample there are still some less-intense halos at 27.900, 40.450, 53.40, and 60.30. If we compare diffraction pattern for blackthorn extract and CuO-NP-bth (Figure 2), we find that both these samples are amorphous. The blackthorn extract is characterized by a very large diffraction peak (halo) around 180, while two new peaks are present in CuO-NP-bth at 21.4 and 33.7. Besides this halo sample, CuO-NP-bth also contains a diffraction peak at the angle 36.6, which belongs to the Cu_2_O compound (PDF:65-3288).

By comparing powder diffraction from bth extract, Cu-Np-bth, and Cu-Np-cel, it is evident that there is a tendency of ordering for amorphous phase because the diffraction peaks become narrower and increase in intensity, and supplementary peaks appear.

#### 3.1.2. Morphological Analysis

**Figure 3:**
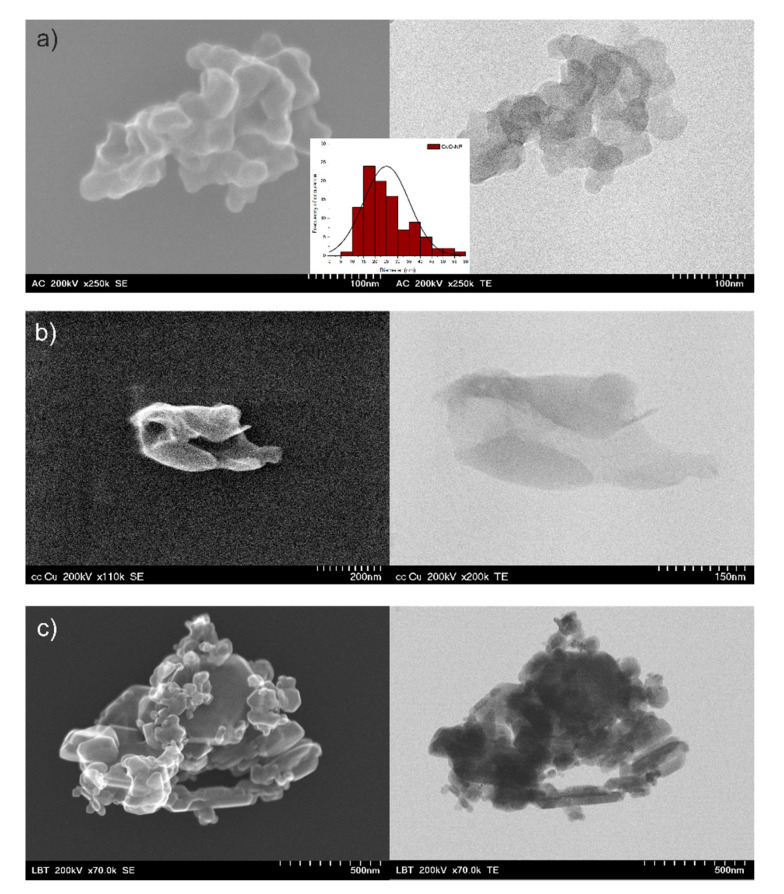
STEM images of green-synthesized CuO-NP using celandine extract and their size distribution (**a**) and blackthorn extract (**b**) and compared to the chemically-synthesized CuO-NPs (**c**).

To establish the morphological characteristics of the NPs, S/TEM analyses were performed. Thus, the CuO NPs synthesized using *C. majus* extract had a polygonal structure and the size varied from 9 nm to 55.8 nm, with an average size of 25 nm ± 1 nm (mean ± standard error of mean, *n* = 100) (Figure 3). The other two types of NP obtained were not distributed as well as CuO-NP-cel, and their size distribution could not be calculated. Other studies using green synthesis also showed similar NP shape and size; for example, Kohatsu et al. [46] showed spherical shape CuO NPs synthesized using green tea extract and Dey et al. [47] showed similar results for CuO NPs synthesized with *Azadirachta indica*.

**Figure 4:**
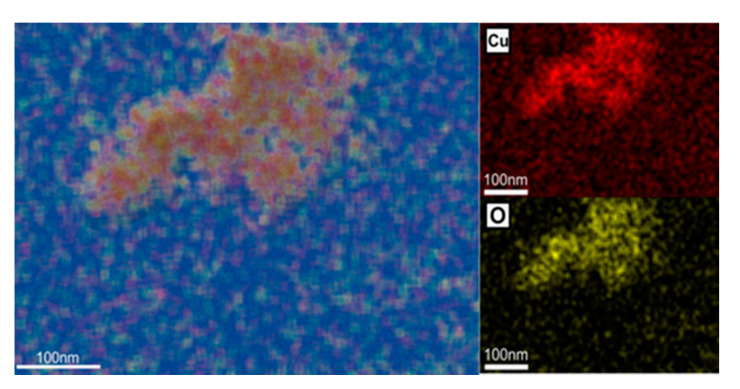
EDX mapping of the elemental distribution in the green-synthesized CuO-NP using *C. majus* extract confirming the CuO formation.

Although it was clear that the formation of NPs occurred, an EDX analysis was performed on the NPs synthesized with *C. majus* extract as they were the most uniformly distributed regarding the morphology. Thus, Cu and O had a uniform distribution (Figure 4), which concluded in the formation of a CuO nanocomplex.

#### 3.1.3. FTIR Analysis

**Figure 5:**
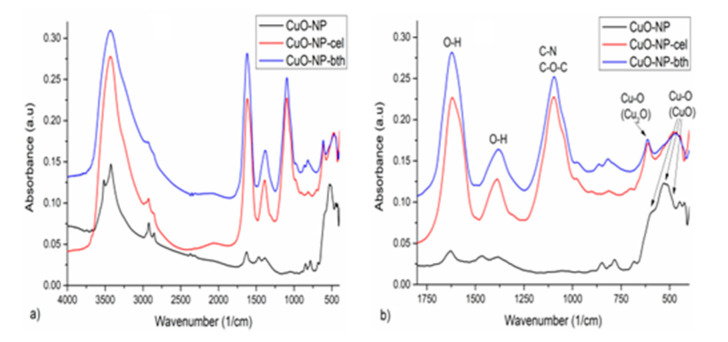
FTIR spectra of the analyzed samples: (**a**) on the entire registered spectral domain, 4000–400 cm^−1^, and (**b**) in the spectral domain of interest, 1750–400 cm^−1^.

The FTIR analysis was performed in order to investigate the structural and molecular bindings between the metal atoms. In the FTIR spectrum of Cu-NP (Figure 5), the characteristic stretching vibration bands of O–H at 3425, 1628, and 1384 cm^−1^, and of C–H at 2923 and 2853 cm^−1^, were identified [48,49,50]. The broad and low-intensity vibration bands from the 700–400 cm^−1^ spectral range, observed at 590 sh, 530, and 484 sh cm^−1^, are characteristic of the stretching vibration of Cu–O from CuO [51].

On the FTIR spectra (Figure 5) of CuO-NP with extracts, some changes occur during the obtaining process, as follows: the stretching vibrations of O–H groups, with high intensity, appear at 3430, 1621, and 1380 cm^−1^, a broad and strong intensity band appears at 1096 cm^−1^ corresponding to C–N group [52], with a shoulder at 1043 cm^−1^ assigned to C–O–C groups, both functional groups appearing due to natural extract [49], and the stretching vibrations of Cu–O from CuO at 534 sh and 482 cm^−1^ can be identified. In both samples a Cu–O vibration at 613 cm^−1^ was observed, which appeared probably due to the reduction of Cu^+2^ to Cu^+1^ (in Cu_2_O) [53,54] in the presence of the extracts.

### 3.2. Analysis of Plant Tissues

#### 3.2.1. Characterization of the Extracts of Assimilating Pigments

**Figure 6:**
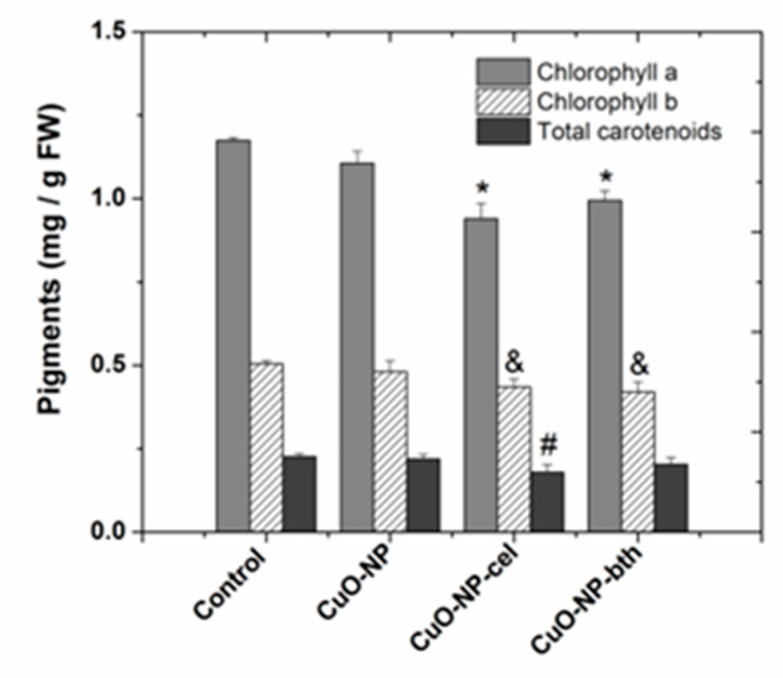
Comparative diagram of chlorophyll a content, chlorophyll b, and total carotenoids. Each data point is the mean ± the standard error of the mean of three independent replicates; the symbols above the columns demonstrate statistically significant differences (*p* < 0.05) between the treatment and the control; “*” statistically significant differences for chlorophyll a, “&” for chlorophyll b, and “#” for carotenoids. FW = fresh weight.

The results obtained from the quantitative determination of assimilating pigments are presented in Figure 6.

For plants grown in the presence of CuO NPs, there was a decrease of about 8–25% in the amount of total chlorophyll a, 10–26% in the amount of total chlorophyll b, and 5–18% in the amount of carotenoids. Carotenoids serve two key roles in plants and algae: they absorb light energy for use in photosynthesis and they protect chlorophyll from photodamage [55]. The high correlation between total carotenoids and chlorophyll could suggest that the protection system of the plant is not damaged.

#### 3.2.2. Determination of Total Phenolic Content

**Figure 7:**
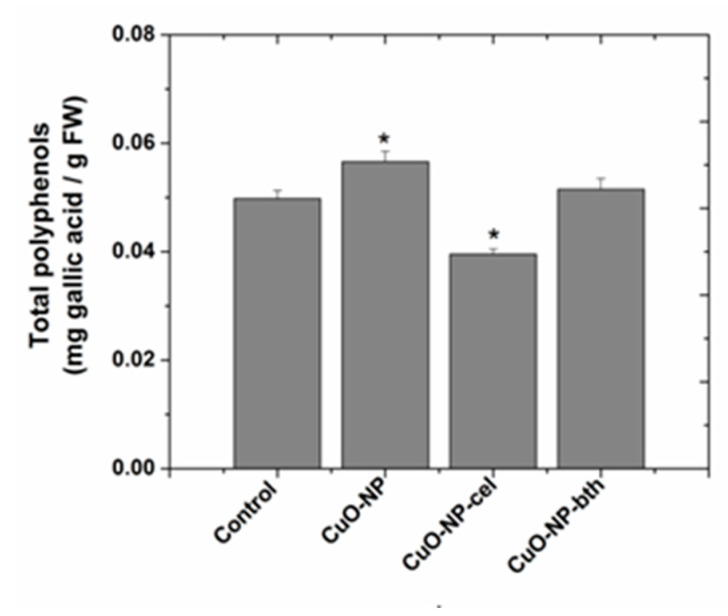
Total polyphenol content expressed as gallic acid equivalents in wheat. Each data point is the mean ± the standard error of the mean of three independent replicates; the symbols “*” above the columns demonstrate statistically significant differences (*p* < 0.05) between the treatment and the control.

The amount of polyphenolic compounds (Figure 7) was expressed as mg gallic acid/g fresh weight (FW). 

Compared to the control plants, the amount of total polyphenols increased in the case of wheat grown in the presence of CuO-NP and CuO-NP-bth and decreased in the case of wheat grown in the presence of CuO-NP-cel.

#### 3.2.3. Determination of Antioxidant Capacity

**Figure 8:**
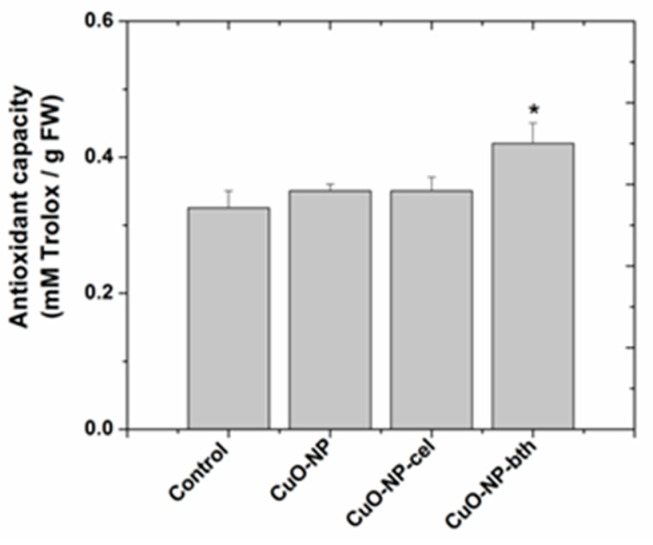
The antioxidant capacity of wheat extracts. Each data point is the mean ± the standard error of the mean of three independent replicates; the symbol “*” above the column demonstrates statistically significant differences (*p* < 0.05) between the treatment and the control.

Since the DPPH method is simple, stable, and reproducible [56], it is often used for antioxidant capacity determination. The antioxidant capacity (Figure 8) was expressed in mM Trolox equivalents (mM Trolox/g sample). 

Plants grown in the presence of CuO NPs showed higher antioxidant capacity compared to control plants. Of the plants grown in the presence of NPs, higher antioxidant capacity was shown in those grown in the presence of CuO-NP-bth. Higher phenolic content in wheat grown on the soil amended with chemically obtained CuO-NP is responsible for bioactivity; therefore, this type of CuO NP is expected to exhibit better results in antioxidant and antibacterial activities. However, the highest antioxidant capacity is shown by the wheat grown in the presence of CuO-NP-bth.

#### 3.2.4. TEM Analysis of Wheat Tissue

**Figure 9:**
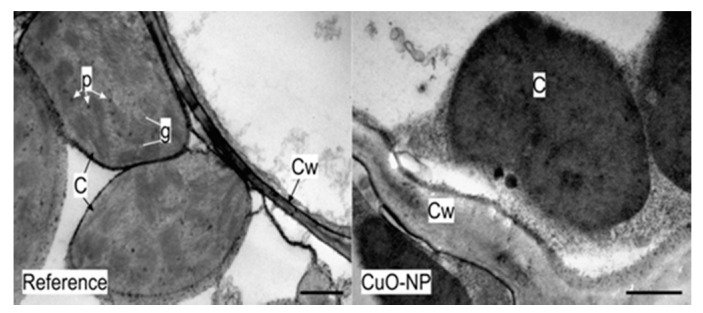
TEM micrographs showing the ultrastructure of the wheat leaves; C = chloroplast, Cw = cell wall, g = grana, *p* = plastoglobule; bars at 1 µm.

To better understand the effects of the obtained NPs at subcellular levels, the leaves of *Triticum aestivum* were analyzed through transmission electron microscopy (TEM). Out of the samples treated with the chemically- and green-synthesized NPs, the leaves of the plants treated with CuO-NP seemed to be affected the most. The major difference occurred in the chloroplasts. There were multiple zones where chloroplasts had no thylakoids, grana, or starch granules, and the stroma had an amorphous distribution (Figure 9). This could result in a poor photosynthetic rate and decreased chlorophyll content. However, the results showed no significant differences between the chlorophyll content of plants treated with synthetic CuO-NP and untreated controls, and further analyses are required to determine if CuO-NPs could affect the photosynthetic rate.

Da Costa and Sharma [57] observed the same effect on *Oryza sativa*, where the chloroplasts were affected and had a lower number of thylakoids. On the Cu-tolerant plant *Elshotlzia splendens*, Shi et al. detected CuO-NP inside the cells of the roots, but also in the leaves, and the plant growth was negatively affected [58]. Various studies confirmed the negative effect of chemically-synthesized CuO-NP on different plants: *Raphanus sativus*, *Lolium perenne*, *Lolium rigidum* [59], *Brassica oleracea*, *Lactuca sativa*, *Medicago sativa* [60,61], *Elodea densa* [62], etc., and also on *Triticum aestivum*. Hafeez et al. [63] showed that in lower concentrations, green-synthesized CuO-NP produced healthier plants, and only high levels of Cu lead to cytotoxicity and DNA damages. On the other hand, other studies proved that chemically-synthesized CuO-NP had an inhibitory effect on the plants’ growth, and the vigor and yield of *Triticum aestivum* was affected [64,65,66,67].

**Figure 10:**
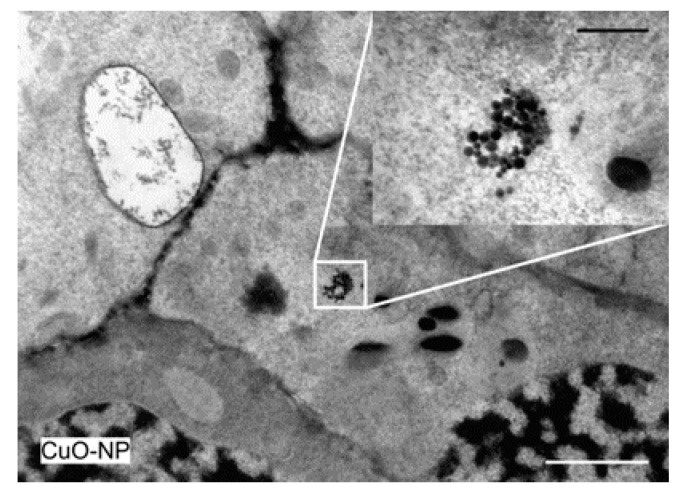
TEM micrograph showing the ultrastructure of the root of chemically-synthesized CuO-NP plants; bar at 2 µm and inset with bar at 500 nm.

The roots of the plants had normal ultrastructure. However, electron-dense accumulations were observed inside the cells of plants treated with chemically-synthesized CuO-NP (Figure 10). Since these were the NPs with the highest contrast as indicated by the S/TEM analysis (Figure 3), they were detectable only in the plants treated with this type of NP. This indicates that the herein-synthesized NPs could penetrate the cell walls of the roots and accumulate in the plants.

**Figure 11:**
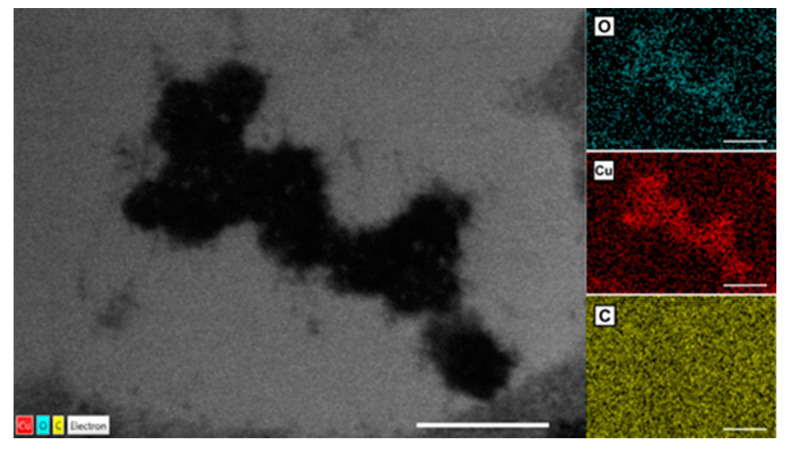
EDX mapping of the electron-dense accumulations inside the cells of the CuO-NP treated plants; bars at 1 µm.

To determine the nature of the electron-dense accumulations, an EDX analysis was performed on the TEM images. This confirmed that the CuO NPs obtained herein are able to penetrate the roots and accumulate in their cells (Figure 11).

#### 3.2.5. PCA Analysis

**Figure 12:**
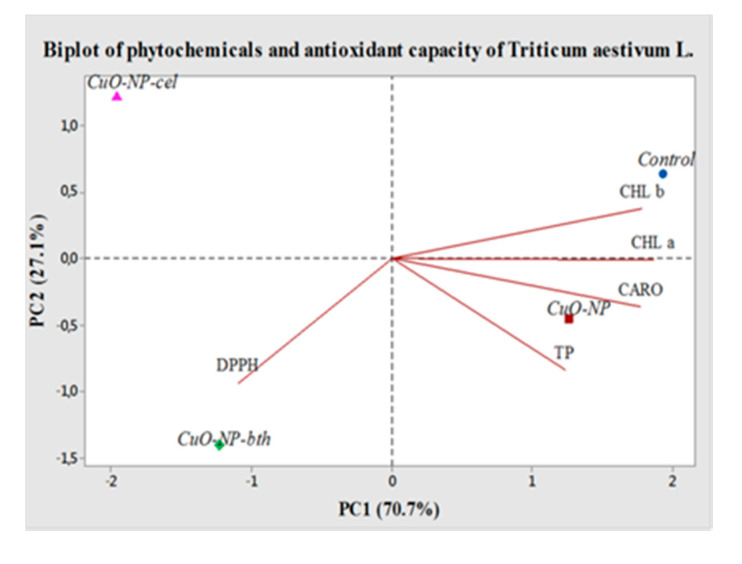
Correlation between the conditions of plant growth, phytochemicals, and antioxidant capacity of wheat. TP: total polyphenols; CHL a: chlorophyll a; CHL b: chlorophyll b; CARO: total carotenoids; DPPH: antioxidant capacity.

PCA multivariate analysis was performed in order to visualize relationships between chlorophyll a, chlorophyll b, carotenoids, polyphenols, and antioxidant activities of studied plants. Thus, in order to reduce the multidimensional structure of the data, the biplot PCA was provided (Figure 12).

The first principal component (PC1) had the highest eigenvalue of 3.53, explaining 70.7% of the total variance (97.8%), and the second principal component (PC2) had an eigenvalue of 1.35, and explains 27.1% of the total variance.

The concentration of each phytochemical with respect to antioxidant capacity increases in the direction of the lines, the parameters with the vectors in the same direction having a positive correlation between them. CHL b was observed with positive loading on the right upper side of the biplot, while CHL a, CARO, and TP were grouped on the right lower side, with only DPPH being observed on the left lower side of the biplot. Thus, PC1 was correlated positively with TP, CHL a, and CARO, while PC2 had high component loadings from the variable analyzed by CHL b and weaker ones by DPPH, whereas it had negative loadings from PF, CHL a, and CARO.

### 3.3. Soil and Plant Element Content

#### 3.3.1. Soil Content and Enrichment Factor in Soil 

In all soil samples, beside the control one, 40 elements were determined. Cu was not detected in control soil. A synthesis of the results is presented in a comprehensive table (Table 1).

Table 1 shows the ranking of element content in each type of investigated soil. The elements that show negative significant difference to the control soil values are presented in blue, and those with positive significant difference within 95% level of confidence in red. The enrichment factor in soil is also presented in Table 1, and discussed below.

There are only a few elements that show the same type of changes for all amended soils: Cl concentration is higher than in the control soil, and Ti and Cs content are lower. On the other side, both soils amended with CuO NP prepared using biological extracts present more similarities: seven elements (Al, Ti, Co, Zn, Br, Cs, and Au) have significant decline in concentration, while Cl and K have a significant positive tendency. Total contents of PTEs in soil follow almost the same ranking in control and exposed soils: Mn > Ba > Zn > V > Cr > Ni > As > Co > Sb > Sn > U > Mo. The first difference consists of the Cu and Ba position in the ranking of exposed soils. In the soil exposed to CuO-NP, the Cu content is higher than Ba, while in the soil exposed to biogenic NPs, the Ba content is higher than that of Cu, but lower than that of Mn. Another difference is the advanced position of Sb in the ranking, immediately after Zn, in the soil exposed to CuO-NP-bth compared to all other soils.

The enrichment factor (EF) of different elements (metals) is an indicator usually used to assess the presence and intensity of anthropogenic contaminant on surface soil pollution studies [68]. This index is calculated by the normalization of one element concentration in the topsoil with respect to the concentration of a reference element. A reference element is an element particularly stable in the soil, which is characterized by absence of vertical mobility and/or degradation phenomena, and its concentration should not be anthropogenically altered. Typical elements used in many studies are Al, Sc, Fe, Mn, and Rb. In our experiment, the EF soil index is used to follow the influence of amending soil with NP obtained by different methods in comparison with the control soil. Al was used as a reference lithogenic element. There is not a well-established classification of EF index. Barbieri et al. [68] suggest that EF < 2 indicates deficiency to minimal enrichment, 2 < EF < 5 is a moderate enrichment, 5 < EF < 20 is a significant enrichment, 20 < EF < 40 is a very high enrichment, and EF > 40 indicates extremely high enrichment. Zhang and Liu [69] use 0.5 < EF < 1.5 to identify trace elements entirely provided from crustal contribution and EF > 1.5 to indicate that an important proportion of trace elements is delivered from non-crustal materials, for example, biota and/or pollution.

The finer discrimination used by Zhang and Liu [69] was applied. Only three elements (Cl, Fe, and Eu) have EF higher than 1.5 for all amended soils. While EF for Fe in the amended soils is very low dispersed (1.58 < EF < 1.81), the EF for Cl and Eu is 4.41 and 3.62 for soil amended with CuO-NP-bth. Besides Cl, Fe, and Eu, the only element with EF > 1.5 for all soils amended with CuO NP is Nd. For CuO-NP-cel: K, Ni, Rb and for CuO-NP-bth: K, Ni, As, Sb, Yb. The highest EF value of 16.20 was obtained for Sb in soil amended with CuO-NP-bth. All other EF values are lower than 1.5, which means that the addition of NP in the control soil does not influence their content in amended soils.

#### 3.3.2. Element Content and Enrichment Factor in Wheat

In the wheat control sample, 34 elements were determined (Na, Mg, Al, Cl, K, Ca, Sc, Cr, Mn, Fe, Co, Ni, Zn, As, Br, Rb, Sr, Zr, Mo, Sb, Cs, Ba, La, Ce, Nd, Sm, Eu, Tb, Tm, Yb, Hf, Ta, Th, and U), but only 14 of them were identified in the exposed wheat samples (Table 2).

A total of 12 elements (Na, Mg, Al, Cl, K, Ca, Mn, Zn, Br, Rb, Sr, and Ba) were determined in all wheat samples, including the control wheat. Another 18 elements (Sc, Cr, Co, Ni, Zr, Cs, La, Ce, Nd, Sm, Eu, Tb, Tm, Yb, Hf, Ta, Th, and U) were identified in control wheat only. Mo, As, and Sb were not detected in samples treated with CuO NP.

Besides the wheat control, Fe was also determined in CuO-NP-bth and Sm in both type of samples treated with CuO NP obtained with plant extracts. Ti, V, Sn, W, and Au were not detected in any wheat samples.

It is interesting to mention that Cu was detected in the wheat treated with CuO-NP only. Kohatsu et al. [46] also reported that Cu content in lettuce leaves was not affected by soil irrigation with solution of biogenic CuO NPs.

Taking into consideration the determination errors, it was determined that Cl, Al, Na, K, Zn, Br, Sr, Ba, and Fe significantly differ in all wheat samples grown on amended soils to the control wheat. The content of Cl, Na, Sr, Fe, and Ba decreased, and Al, K, Zn, and Br increased. In the samples grown on the soil amended with CuO NP obtained with plant extracts, three elements (As, Sb, and Sm) had a significant decrease and Mg had a significant increase. Fe also decreased in wheat treated with CuO-NP-bth. A significant decrease in Sb content and increase in K, Zn, and Mg content in lettuce leaves were also reported by Kohatsu et al. [46], while the influence of soil irrigation with solutions of biogenic CuO NP was studied. As content was not affected by exposure, and Na and Ba contents were significantly increased. The elements whose contents decreased are presented in blue in Table 2 and those with an increase in content in red.

The literature offers information about investigations performed with metal NPs on wheat samples such as Ti, Cu, Zn, Ce, and Ag. The majority of them were focused on the accumulation, uptake, and effect of Ti NPs on the growth of root and shoot [70,71,72,73,74], on the performance of wheat in the presence of CuO and ZnO in nano and non-nanometric forms [64] and nAl_2_O_3_ [75], the effect on seed germination [63,76], and toxicity of CeO_2_ NPs [77,78]. There are no studies following the modification of the total elemental content in wheat while the samples are treated with some NPs. This fact can be connected with the lack of information concerning the elemental content in wheat plants.

Data on concentration of Se, Pb, and Mn in several plant species, including wheat, collected in Iran were reported by Sakizadeh et al. [79,80].

In the study performed by Mishra et al. [81], concerning the element uptakes by root, stem, leaf, and seed of wheat grown in graded levels of municipal solid waste-amended soils, the data on the control content of Ni, Zn, Cu, Cd, Cr, and Pb are presented.

Additional information concerning the control level of Zn in natural untreated wheat is reported by Munir et al. [82] within the study of the effect of ZnO NPs on the growth and uptake in wheat.

Data on Fe, Zn, Mn, Cu, Ni, and Cd were reported for wheat grown under low-N and high-N fertilization rates and over two growing seasons [83], but unfortunately no data for wheat grown in unamended soil exists in this study.

A larger spectrum of elements, including Sr, Rb, and Mo, was determined by Suchowilska et al. [84] in wheat seeds, and it was proven that the concentrations of the investigated elements are a species–specific character.

In order to understand the soil–plant relationship, the enrichment factor to the substrate of each sample was calculated. Aluminum content was chosen as the lithogenic element. The majority of EFs have values lower than 1.5, which means that their content is not affected by the amended soil content used for their growing. However, K and Br have much higher EF for all wheat samples. It is important to mention that in the case of K, all wheat samples grown on amended soils presented a high mobility ratio, indicating very significant accumulator behavior. A clear correlation between mobility ration and enrichment factor was not observed in the case of Br. Mo presented increased EF for the wheat samples grown on soil amended with plant extract-based CuO NP. All these values are higher than 7, while the mobility ratio is about 1 and indicates an indifferent behavior

#### 3.3.3. Soil to Plant Transfer

Another approach to assess the element soil to plant transfer is to calculate the mobility ratio [85]. Baker [86] showed that the plants have different strategies in interaction with heavy metals. They can act similar to accumulators (MR > 1) or excluders (MR < 1), or they can have an indifferent behavior to some elements (MR~1). The authors appreciated that a 20% variation about 1 (from 0.8 to 1.2) can be considered relevant for an indifferent behavior.

In our experiment, all samples, including the control, behaved similar to excluders of Na, Mg, Al, Ca, Mn, As, Sr, Sb, and Ba. There are several elements (Sc, Cr, Co, Ni, Zr, La, Ce, Nd, Sm, Tb, Tm, Hf, Ta, Th, Yb, and U) which were determined in the control plant only. Control wheat accumulated the first 14 elements in enumeration, excluded Yb, and was indifferent to U (Table 3).

All plant samples had a very high accumulator behavior for Cl 4.28 < MR < 34.7. The highest value belongs to the control wheat, and the lowest to the wheat grown on the soil amended with CuO-NP-bth.

In the case of K, all wheat samples grown on amended soils had very significant accumulator behavior, while the control wheat seems to be indifferent to K.

The control wheat accumulated Fe, but the wheat samples grown on amended soils behaved similar to excluders (CuO-NP-bth) of Fe. In the wheat samples treated with CuO-NP-cel, the Fe content was under the detection limit.

The wheat control behaved similar to an excluder for Ti, while wheat samples treated with CuO NP obtained with plant extracts again had different behavior: accumulator and indifferent to it for blackthorn and celandine extracts, respectively.

The control wheat excluded Br, while wheat treated with CuO-NP-bth was indifferent to it, but wheat treated with CuO-NP-cel had evident accumulator behavior up to MR > 8.

The control wheat, together with wheat treated with CuO-NP-bth, accumulated Rb while wheat treated with CuO-NP-cel excluded it. At the same time the MR values for all these samples were very low, spreading from 0.76 up to 1.54.

The wheat samples treated with both plant extract-based CuO NP were indifferent to Mo and the control wheat excluded it. Cs was detected in wheat treated with CuO-NP-cel and control only. The control wheat was indifferent to Cs, while wheat treated with CuO-NP-cel excluded it.

It is obvious that both processes of exclusion and accumulation of the chemical elements in wheat plants grown on amended soils are less active than in the control wheat; thus, it can be concluded that the addition of CuO NP in soil influences it. Soil pH influences solubility, concentration in soil solution, ionic form, and mobility of micronutrients in soil and, consequently, acquisition of these elements by plants [87]. As a rule, the availability of B, Cu, Fe, Mn, and Zn usually increases, and Mo decreases, as soil pH decreases. Our study was performed on a slightly alkaline substrate with a pH of 7.4.

Despite the fact that it was proved that total K/exchangeable K ratio varies depending on the location and depth of the studied soil sample [88], there is a positive correlation between total content of K in soil and its exchangeable part [89] that is accumulated by plants. Taking into consideration that the K mobility factor is higher than 1.2 for all plants grown on amended soils, while the control is indifferent to K, it can be supposed that the use of NPs could increase the mobility of K in substrate.

While calcium (Ca^2+^), magnesium (Mg^2+^), and potassium (K^+^) are considered basic cations, the most used criteria for estimating the soil quality are Ca:Mg, Ca:K, and Mg:K ratios. These ratios vary in quite wide intervals and present as follows: a Ca:Mg of 5.4–14.2:1, Ca:K of 13–42.5:1, and Mg:K of 1.2–6:1, if referring to the concept of “ideal” soil developed by McLean [90] and McLean and Brown [91] in 1984 as an upgrade of the preliminary version suggested by Bear et al. in 1945 [92].

In our study, the mobility factor for Ca is very close to zero for all plants, which suggests that the Ca mobility in soil was not influenced by the addition of the NPs.

In the case of Mg, it is not so easy to conclude the effect of amending soil to the element mobility in substrate, because the mobility factor for plants grown with CuO-NP-cel is slightly higher than 1.2, while that for the plants grown with CuO-NPs is much higher. The control plants and those grown with CuO-NP-bth have mobility factors close to zero and 1, respectively.

A similar situation occurred with the Zn mobility. The Zn mobility coefficient increased from less than 0.5 up to 0.89, 1.2, and 1.24 for plants grown with CuO-Np, CuO-NP-cel, and CuO-Np-bth, respectively.

With regard to chlorine, it can be said that the mobility factor is significantly reduced compared to the control for all samples. The highest (eightfold) decline was observed for plants grown with CuO-NP-bth, while the decline for the other plants was also high, but only about threefold. It seems that the use of CuO-NPs inhibits Cl mobility.

### 3.4. Correlation between the Element Content and Phenolic Compound Concentration, Assimilating Pigments Concentration, and Antioxidant Capacity in Plants

Since it was proved that the chlorophyll content can be considered as a measure of plant health [93], in order to check the potential relationship between the element content and plant health and its resistance to environmental factors, the correlation coefficient between the element content and TP, CHL a, CHL b, CARO, and DPPH was calculated (Table 4).

The use of biogenic NPs led to an increase in Mo concentration, an increase which is in strong anticorrelation with the level of CHL a (R^2^ = −0.99), CHL b (R^2^ = −0.96), and CARO (R^2^ = −0.90). Al, Mg, K, Zn, and Ca present a similar relationship, but less pronounced, with less-negative correlation coefficients. All other determined elements were partially released from the plant when NPs were applied, and are correlated with the levels of CHL a, CHL b, and CARO.

DPPH showed absolutely opposite behavior in relation to Al, K, Zn, and Mg. The increase of the content of these elements occurs with the increase of antioxidant capacity. The Ca content is tightly correlated with DPPH values (R^2^ = 0.93).

As for TP, it represents a moderate correlation (R^2^ = 0.67) with the behavior of Br and a weak anticorrelation with the behavior of Al (R^2^ = −0.43). Other elements have a neutral connection with TP.

In plants, relatively little is known about the Cu transport into and within cells showing a dependence on Cu for Fe, Zn, Mn, and other element assimilation [94]. Cu–Fe and Cu–Zn antagonism often occurs in plants grown under Cu toxicity [95,96,97]. Nevertheless, the opposite scenario has been also observed in several plants exposed to Cu toxicity in soil. An increasing concentration of soil Cu resulted in a parallel increase in leaf Cu content with no reduction in the leaf of other metals [98]. Soybean plants exhibited Zn content increase upon Cu treatment through roots [99].

In our study, the results of Zn show the same trend; the Zn content increases when delivered through the soil but the Zn values fall within the sufficiency range for wheat [100] and do not exceed the normal Zn range in most plants, which varies between 50 to 100 ppm [101,102].

Although required by plants in small amounts, Fe is involved in many important compounds and physiological processes in plants. Fe’s involvement in chlorophyll synthesis is the reason for the chlorosis (yellowing) associated with Fe deficiency. In our study, the Fe value in the control sample is very high, almost the same as in the soil, and this may indicate contamination of samples with soil, but the Al values do not increase. A more detailed analysis of this aspect is necessary. The Cu–Fe antagonism was also present in our study; Fe was practically released from plants under the influence of CuO-NP. After applying CuO-NP, Fe was detected only in wheat grown with CuO-NP-bth, and the value fell in the sufficiency range [100].

Because of its mobility, the K content in plants varies widely depending on the type of plant, the age of the plant, and even the amount of precipitation. The K content in the control was lower than in the reference plant [101], and in the lower boundary of the sufficiency range [99], while the values in wheat grown with NP are about three times higher than the upper boundary of the sufficiency range.

Magnesium is the second-most abundant cation in plants after K. The most widely studied aspect of Mg activity in plants is the role it plays in photosynthesis. Optimal plant growth requires 1.5–3.5 g of Mg per kilogram of dry matter for many physiological and biochemical processes [100,102,103,104]. Our results of 1.8 g/kg for control plants and ~2.0 g/kg for plants grown on amended soils are within the specified range. The Mg content in plants grown on altered soils is significantly higher than in the control, but, on the other hand, if the mobility coefficient is taken into account, the plant behaves as an Mg excluder.

Along with K, Zn, Mg, and Fe, Mo is considered an essential element in plants. Molybdenum deficiency occurs in many plants when the plant concentration is less than 0.10 ppm. The Mo value in our control sample is just at the border of deficit, while the samples grown on soil amended with CuO-NP-cel and CuO-NP-bth have much higher values, but not exceeding the critical value for forage (15 mg/kg) [97] and corresponding to the values suggested by Markert [101] for the “reference plant”. Molybdenum was not detected in the plants grown on soil amended with CuO-NP.

Aluminum is not considered a plant nutrient because plants do not require it. However, its presence in plants can affect the normal function of some other elements. Aluminum levels in excess of 400 ppm in young tissue or 200 ppm in mature plants and leaves are undesirable [105]. Our results show lower Al values for all samples and are in the same magnitude as the Markert’s reference plant.

Calcium is an essential plant nutrient. Plants growing with adequate Ca in their natural habitats have shoot Ca concentrations between 0.1 and 5% d. wt. [98]. Calcium competes with other positively charged ions, such as sodium (Na^+^), potassium (K^+^), and magnesium (Mg^2+^). In our study, the content of Ca in plants is close to the lower limit mentioned by White and Broadley [106] and lower than the values suggested by Markert for the “reference” plant.

## 4. Conclusions

In analyzing the plants exposed to CuO NPs, it was found that the amounts of chlorophyll and carotenoids decreased, while the amount of polyphenols and the antioxidant capacity increased, compared to the control group. Regarding bioactive compounds, the most affected plants were those grown in the presence of CuO-NP-cel.

In addition, the ultrastructural analysis revealed that several changes occurred in the leaves of the treated plants, compared to the controls. All wheat samples exposed to chemical or biogenic CuO NPs showed changes in elemental accumulation in plant tissues. Soil amending completely inhibits the accumulation of seventeen elements, regardless of the type of NPs applied. Exposure to chemically obtained CuO NPs led to a more obvious alteration of the element profile compared to control and biogenic CuO NPs. Application of CuO NPs caused the accumulation of K, Br, Al, and Zn and the decrease of Cl, Na, Ba, and Sr in wheat samples, regardless of the type of NPs applied. The application of chemically obtained NPs induced the most significant changes by blocking the assimilation of Fe, Mo, As, Sb, and Sm and favoring much higher accumulation of Br than biogenic NPs. The decrease in chlorophyll and carotenoid contents is correlated with an increase in the antioxidant capacity and with the increase of Mo, Al, Mg, K, Zn, and Ca elements. The behavior of total polyphenols is correlated with Br content and is antagonist to Al behavior.

Considering the obtained results, it can be concluded that CuO NPs have a negative influence on ultrastructure of wheat plants, which is reflected in the level of production and quality.

## Figures and Tables

**Table 1:** Ranking of element content in each type of investigated soils and significant enrichment factors.

Parameter	Control Soil	Soil with CuO-NP	Soil with CuO-NP-cel	Soil with CuO-NP-bth	Remarks
Elements determined in soil	Ca > Mg > Al > Fe > K> Na > Ti > **Mn** > Cl > **Ba** > Sr > **Zn** > Zr > **V**>Rb> **Cr** > Ce > Br > La > **Ni**> Nd > **As** > **Co** > Sc > Th > **Sb** > W>Hf > Sm > Cs > **Sn** > **U** > **Mo** > Yb > Ta > Au > Tb > Tm > Eu	Ca > Mg > Al > Fe > K > Na > Ti > Cl > Mn > **Cu** > Ba > Sr > **Zn** > Zr > Rb > V > Cr > Ce > Br > **Ni** > La > Nd > As > Co > Sc > Sb > Th > W > Hf > Sn > Sm > Cs > U > **Mo** > Yb > Ta > Tb > Eu > Tm > Au	Ca > Al > K > Mg > Fe > Na > Ti > Cl > **Mn** > **Ba** > **Cu** > Sr > Zn > Zr > Rb > **V** > **Cr** > Ce > Nd > **Ni** > La > Br > As > Co > Sc > Th > Sb > Hf > **Sn** > Sm > Cs > W > U > **Mo** > Yb > Tb > Ta > Eu > Tm > Au	Ca > Mg > Al > Fe > K > Na > Cl > Ti > **Mn** > **Ba** > **Cu** > Sr > Zr > Zn > Sb > Rb > V > **Cr** > Ce > **Ni** > Nd > Br > La > **As** > Co > Sc > Th > Hf > Sm > Cs > **U** > Yb > W > **Mo** > Ta > Eu > Tb > Tm > Au	Cl always higher Ti and Cs always lower
EF/Al soil		EF (Cl, Fe, Eu, Nd) > 1.5	EF (Cl, Fe, Eu, K, Ni, Rb) > 1.5	EF (Cl, Fe, Eu, K, Ni, As, Sb, Yb) > 1.5	EF (Cl, Fe, and Eu) > 1.5 for all amended soils

In blue: elements with a decrease in concentration with regard to control value: in red: those in which content increased. In bold: potentially toxic elements (PTEs); EF/Al—enrichment factor with Al as reference element in soil.

**Table 2:** Ranking of element content in each type of investigated plant, and significant enrichment factors.

Parameter	Control Soil	Soil with CuO-NP	Soil with CuO-NP-cel	Soil with CuO-NP-bth	Remarks
Elements determined in plants	Cl > Fe > K>Ca > Mg> Na > **Ba** > Zr > Al > **Cr**> Rb > **Zn** > **Ni** > Sr > Ce > **Mn** > **La** > Nd > **Co** > Sc > Th > **As** > Hf > Sm > Cs > U>Br > **Sb** > Ta > Eu> Tb > Yb > Tm > **Mo**	K > Cl > Ca > Mg > Br > Na > Al > Zn > Rb > **Cu** > **Mn** > Ba > Sr	K > Cl > Ca > Mg > Na > Al > Zn > Rb > **Mn** > Ba > Sr > Br > **Mo** > As > Sb > Sm	K > Cl > Ca > Mg > Na > Al > Fe > Zn > Rb > **Mn** > Ba > Br > Sr > **Mo** > As > Sb > Sm	
EF/Al wheat		K, Br >> 1.5	K, Br, Mo >> 1.5	K, Br, Mo >> 1.5	

In blue: elements with a decrease in concentration with regard to control value; in red: those in which content increased. In bold: potentially toxic elements (PTEs); EF/Al—enrichment factor with Al as reference element in wheat.

**Table 3:** Plant behavior in interaction with chemical elements.

Parameter	Control Soil	Soil with CuO-NP	Soil with CuO-NP-cel	Soil with CuO-NP-bth	Remarks
Soil to plant mobility ratio (MR)	Excl.: Na, Mg, Al, Ca, Mn, As, Sr, Sb, Ba, Yb, Ti, Br, Mo Accum.: Sc, Cr, Co, Ni, Zr, La, Ce, Nd, Sm, Tb, Tm, Hf, Ta, Th, Cl, Fe, Rb Indiff.: U, K, Cs	Excl.: Na, Mg, Al, Ca, Mn, As, Sr, Sb, Ba Accum.: Cl, K, Br Indiff: Zn	Excl.: Na, Mg, Al, Ca, Mn, As, Sr, Sb, Ba, Rb, Cs, Sm Accum.: Cl, K, Br Indiff.: Ti, Mo, Zn	Excl.: Na, Mg, Al, Ca, Mn, As, Sr, Sb, Ba, Fe, Sm Accum.: Cl, K, Ti, Rb, Zn Indiff.: Br, Mo	MR_Cl_: control >> chem > cel >> bth

Excl.—plants act similar to excluders with regard to elements (MR < 1); Accum.—accumulator behavior (MR > 1); Indiff.—indifferent behavior (MR~1); MR_Cl_—soil to plant mobility ration for Cl.

**Table 4:** Content of bioactive compounds compared to control, and correlation with elemental content.

Parameter	Control Soil	Soil with CuO-NP	Soil with CuO-NP-cel	Soil with CuO-NP-bth	*Remarks*
CHL a		<control	<control	<control	*chem > bth > cel*
CHL b		<control	<control	<control	*chem > cel > bth*
CARO		<control	<control	<control	*chem > bth > cel*
TP		>control	<control	>control	*chem > bth > cel*
DPPH		>control	>control	>control	*chem~cel < bth*
R^2^	R^2^ (C_Mo_-CHL a) = −0.99; R^2^ (C_Mo_-CHL b) = −0.96; R^2^ (C_Mo_-CARO) = −0.90, R^2^ (C_Al,Mg, K, Zn, Ca_ –CHL a, CHL b, CARO) < 0 R^2^ (C_Al, Mg, K, Zn_ –DPPH) = 0.93 R^2^ (C_Br_ –TP) = 0.67; R^2^ (C_Al_ –TP) = −0.43				

TP—total polyphenols, CHL a—chlorophyll a, CHL b—chlorophyll b, CARO—total carotenoids, DPPH—antioxidant capacity; R^2^ = correlation between elemental content and TP, CHL a, CHL b, CARO, and DPPH.
